# Targeting the FTO-ACSL4 Pathway: A Novel Mechanism for Sanguinarine Chloride-Induced Ferroptosis in Endometrial Cancer

**DOI:** 10.3390/biomedicines14030608

**Published:** 2026-03-09

**Authors:** Wenyan Li, Shanhui Liu, Ke Wang, Jianzhong Lu, Shengjun Fu, Lanlan Li, Yan Tao

**Affiliations:** 1 Department of Gynaecology and Obstetrics, The Second Hospital of Lanzhou University, No. 82 Cuiyingmen, Lanzhou 730030, China; liwenyan20260112@163.com; 2 Institute of Urology, Gansu Province Clinical Research Center for Urinary System Diseases, The Second Hospital of Lanzhou University, No. 82 Cuiyingmen, Lanzhou 730030, China; liushh2014@lzu.edu.cn (S.L.); lujzh@lzu.edu.cn (J.L.); fubeta@163.com (S.F.); llli12@lzu.edu.cn (L.L.); 3 The Second Hospital & Clinical Medical School, Lanzhou University, Lanzhou 730030, China; 320220907550@lzu.edu.cn

**Keywords:** endometrial cancer, sanguinarine chloride, ferroptosis, FTO, ACSL4

## Abstract

**Objective:** Endometrial cancer (EC) remains a significant clinical challenge, particularly for patients with advanced or recurrent disease. This study aims to investigate the effects of Sanguinarine Chloride (S.C), a natural benzophenanthridine alkaloid with broad anti-tumor properties, on EC cell growth and invasion, and to elucidate its underlying molecular mechanisms. **Methods:** S.C’s effects on EC cell viability, proliferation, invasion, and apoptosis were evaluated using CCK-8, EdU, colony formation, 3D matrigel drop assay, FACS and Western blotting (WB). To evaluate its effects on ferroptosis, malondialdehyde (MDA) assay kits, DCFH-DA and the C11 BODIPY581/591 probe, were employed. The molecular mechanisms through which S.C regulates FTO-ACSL4 axis were investigated using plasmid transfection and WB. Additionally, a mouse xenograft model derived from EC cells was established to evaluate the in vivo effects of S.C and its molecular mechanisms, utilizing hematoxylin and eosin (H&E) staining, immunohistochemistry (IHC) and WB. **Results:** S.C significantly inhibited EC cell growth and invasion. It induced cell death primarily through ferroptosis, as inhibitors (Ferrostatin-1, Deferoxamine) reversed this effect. S.C downregulated the RNA demethylase FTO, leading to increased ACSL4 expression, enhanced lipid peroxidation, suppression of the NRF2-GPX4 axis, and activated NCOA4-mediated ferritinophagy. Knocking down or pharmacologically inhibiting ACSL4 reduced S.C-induced ferroptosis. Furthermore, in a murine xenograft model, S.C significantly suppressed tumor growth, which was associated with consistent alterations in these ferroptosis-related markers in vivo. **Conclusions:** Our findings reveal that S.C triggers ferroptosis in EC via the novel FTO-ACSL4 axis, highlighting its potential as a therapeutic agent and identifying the FTO-ACSL4 pathway as a promising target for endometrial cancer treatment.

## 1. Introduction

Endometrial cancer (EC) ranks among the most prevalent malignancies in the female reproductive system, with its global incidence showing an upward trend [1,2]. Although patients diagnosed at an early stage can achieve favorable outcomes through surgery and adjuvant therapies, those with advanced or recurrent disease continue to face limited treatment options and poor survival rates [3,4]. Therefore, developing novel therapeutic strategies to inhibit the progression and metastasis of endometrial cancer has become an urgent research priority.

The regulation of cell death mechanisms plays a critical role in cancer therapy. Beyond apoptosis and necroptosis, ferroptosis—an iron-dependent form of regulated cell death driven by lipid peroxidation—has attracted significant attention in recent cancer research [5,6,7,8]. Ferroptosis can be triggered by various intracellular events, including mitochondrial dysfunction, accumulation of reactive oxygen species (ROS), dysregulation of antioxidant systems, and metabolic reprogramming of fatty acids [9,10]. Among key regulators, acyl-CoA synthetase long chain family member 4 (ACSL4), a critical enzyme involved in the synthesis of polyunsaturated fatty acid-containing phospholipids, plays a central role in promoting lipid peroxidation and ferroptosis. Growing evidence indicates that targeting the ferroptosis pathway can effectively enhance therapeutic responses in multiple cancer types, highlighting its broad potential in oncology treatment [11,12].

Sanguinarine chloride (C_20_H_14_ClNO_4_) is a benzophenanthridine alkaloid derived from the roots of *Sanguinaria canadensis* (bloodroot) and other plants in the Papaveraceae family [13]. It has been reported to exhibit broad biological activities, including antimicrobial, anti-inflammatory, and antioxidant properties [13,14]. Furthermore, Sanguinarine Chloride has also been demonstrated to exhibit antitumor activity against a variety of cancers, such as breast, gastric, colorectal, and lung cancer [15,16,17,18]. Recent studies further indicate that S.C can promote ferroptosis in multiple types of tumor cells, including prostate cancer, non-small cell lung cancer, cervical cancer, and colorectal cancer [13,19,20,21]. However, its role and underlying molecular mechanisms in endometrial cancer remain largely unexplored. This study aims to systematically evaluate the antitumor effects of S.C in endometrial cancer and investigate whether it exerts its function through the induction of ferroptosis.

Through in vitro and in vivo experiments, we found that S.C significantly inhibits the proliferation, migration, and invasion of endometrial cancer cells, while also inducing mitochondrial dysfunction and cell death. Further mechanistic studies revealed that S.C downregulates fat mass and obesity-associated protein (FTO) expression, upregulates ACSL4, activates nuclear receptor coactivator 4 (NCOA4)-mediated ferritinophagy, and suppresses the nuclear factor erythroid derived 2-like 2/glutathione peroxidase 4 (NRF2-GPX4) antioxidant axis, ultimately leading to mitochondrial lipid peroxidation and ferroptosis. Moreover, both ACSL4 knockdown and inhibition of its activity by berberine reversed the ferroptotic cell death induced by S.C. In a murine xenograft model, S.C similarly exhibited significant tumor-suppressive effects, accompanied by alterations in ferroptosis-related markers.

Our study is the first to demonstrate that S.C triggers ferroptosis via the FTO-ACSL4 axis, thereby suppressing endometrial cancer progression. These findings provide new insights into the molecular mechanisms of S.C and suggest a potential therapeutic strategy for targeting ferroptosis in endometrial cancer.

## 2. Materials and Methods

### 2.1. Materials

Sanguinarine chloride (T0129), Berberine (T4S0797), RSL3 (T3646), Ferrostatin-1 (T6500) and Deferoxamine Mesylate (T1637) were purchased from Targetmol (TargetMol, Boston, MA, USA). CCK8 reagent (CYT001) was purchased from Yoche (Yoche, Shanghai, China). Lipid Peroxidation MDA Assay Kit (S0131S), Annexin V-FITC Apoptosis Detection Kit (C1062L), DCFH-DA (S1105M), Mito-Tracker Red CMXRos (C1035) and RhoNox-6 (S1070M) were purchased from Beyotime (Haimen, China). Matrigel (356234) was obtained from CORNING (Hong Kong, China). BODIPY™ 581/591 C11 (D3861) was sourced from Thermo Fisher Scientific (Shanghai, China). The primary antibodies used for IHC and Western blotting were the following: GAPDH (ab128915), and BAX (ab32503) were purchased from Abcam (Cambridge, UK). SLC7A11/xCT (A2413), GPX4 (A1933), ACSL4 (A20414), SLC40A1 (A14885), FTH (A19544), FTL (A1768), FSP1 (A12128), DMT1/SLC11A2 (A10231), SCD1 (A25168), ALKBH5 (A11684), METTL3 (A19079), USP15 (A6786), FTO (A3861), SENP1 (A4460), TRIM21 (A13547), KEAP1 (A21724) and Ki67 (A21861) were purchased from Abclonal (Wuhan, China). BCL2 (2872), PARP (9532) and cleaved-caspase3 (9664S) were purchased from Cell Signaling Technology (Danvers, MA, USA). NRF2 (16396-1-AP) were purchased from Proteintech (Wuhan, China). NCOA4 (DF4255) was purchased from Affinity (Changzhou, China).

### 2.2. Cell Lines and Cell Culture

The endometrial cancer cell lines HEC-1-A, AN3CA and KLE were obtained from Servicebio (Wuhan, China). HEC-1-A, AN3CA and KLE cells were cultured in McCOY’s 5A or Dulbecco’s modified Eagle’s medium supplemented with 10% fetal bovine serum, 100 U/mL penicillin and 0.1 mg/mL streptomycin at 37 °C with 5% CO_2_.

Cell lines differ in their histological grade and origin of metastasis. HEC-1-A cells were moderately differentiated and originate from the epithelial layer of the endometrium. AN3CA and KLE were poorly differentiated cell lines of endometrial cancer, with AN3CA originating from lymph node metastasis [22]. All cell lines were authenticated by the supplier via short tandem repeat (STR) profiling before distribution. To ensure experimental consistency, cells were expanded and cryopreserved within 2–3 passages of receipt. Regular monitoring for mycoplasma contamination was performed using a PCR-based detection kit (G1900, Servicebio, Wuhan, China).

### 2.3. Plasmid Construction and Transfection

The plasmid pcDNA3.1-FTO was obtained from Wuhan Miaoling Biotechnology Co., Ltd. (Wuhan, China). Lentiviral vectors for ACSL4 knockdown (pLKO.1-ACSL4-1/2/3) were also supplied by the same company. The shRNA target sequences used were as follows: sh-ACSL4-1, 5′—attgctatgatgcatcatcactccc—3′; sh-ACSL4-2, 5′—aagctgaaatactgaaagaaa—3′; sh-ACSL4-3, 5′—tccggaaatcatggatagaat—3′. HEC-1-A and AN3CA cells were transduced with lentiviral supernatants in the presence of polybrene for 12 h. Following infection, the cells were selected under puromycin (8 μg/mL) for two days.

### 2.4. Cell Viability Assay

Cell viability was measured by CCK-8 method. Briefly, cells were seeded into 96-well plates at a density of 5 × 10^3^ cells/well and incubated at 37 °C with 5% CO_2_ for 24 h. Subsequently, cells were treated with different concentrations of S.C, Ferrostatin-1 (Fer-1), or Deferoxamine (DFO) for varying durations. After treatment, CCK-8 reagent was added to each well, followed by incubation at 37 °C for 2–4 h. The absorbance at 450 nm was then measured using a microplate reader.

### 2.5. Clonogenic Assays

The colony-forming unit (CFU) assay was performed to evaluate the effect of S.C on the clonogenic survival of HEC-1-A and AN3CA cells. In brief, cells were seeded into 6-well plates at a density of 500 cells/well for 24 h. Subsequently, cells were treated with different concentrations of S.C. The culture medium and S.C were replenished every two days over a ten-day period. After the incubation, the resulting colonies were fixed with 4% paraformaldehyde and stained with crystal violet solution.

### 2.6. 3D Matrigel Drop Invasion Assay

The 3D Matrigel drop invasion assay was conducted following a previously described protocol [23]. Briefly, 5 × 10^4^ HEC-1-A or AN3CA cells were suspended in 10 μL of matrigel and pipetted as a droplet into a 24-well plate. The droplet was allowed to solidify for 20 min before adding culture medium or S.C. The cells were then cultured for 6 days, with the medium—containing 10% FBS and the respective compounds—replaced once on day 3. On day 6, cell invasion was assessed by measuring the distance cells migrated from the edge of the matrigel drop.

### 2.7. ROS Measurement

Intracellular ROS levels were assessed with the DCFH-DA fluorescent probe following previously described methods [24]. Briefly, after seeding cells in 6 cm dishes and treating them with various concentrations of S.C for 48 h, the culture medium was removed. The cells were then incubated with DCFH-DA at a final concentration of 10 μM for 30 min in the dark. Fluorescence intensity was subsequently measured using a flow cytometer (BECKMAN CytoFLEX, Brea, CA, USA).

### 2.8. Annexin V-FITC/Propium Iodide Assay

HEC-1-A and AN3CA cells were seeded in 60 mm dishes at a density of 5 × 10^5^ cells/well for 24 h. Subsequently, cells were treated with either DMSO or S.C for 48 h. Death rates were then assessed using an Annexin V-FITC apoptosis detection kit (C1062L, Beyotime, Haimen, China) according to the manufacturer’s instructions. The percentage of apoptotic cells was quantified by flow cytometry (BECKMAN CytoFLEX).

### 2.9. Western Blotting (WB) Assay

Cell lines were treated in vitro under the specified conditions and lysed using cell lysis buffer (P0013B, Beyotime, Haimen, China) supplemented with a protease/phosphatase inhibitor cocktail (5872, Cell Signaling Technology, Danvers, MA, USA). The protein concentration of the lysates was determined using a bicinchoninic acid (BCA) assay kit (Beyotime, Haimen, China). Subsequently, the supernatants were separated by 10% or 12% SDS-PAGE and transferred onto 0.45 μm PVDF membranes (10600023, Amersham, Buckinghamshire, UK) for detection of the target proteins.

### 2.10. Malondialdehyde (MDA) Assay

HEC-1-A and AN3CA cells were seeded in 100 mm dishes and treated with either DMSO or S.C for 48 h. Subsequently, the level of MDA was quantified using the Lipid Peroxidation MDA Assay Kit (S0131S, Beyotime Biotechnology, China).

### 2.11. Mitochondrial Staining

Mitochondrial staining was performed using MitoTracker CMXRos (C1049B, Beyotime, Haimen, China) according to the following procedure: The culture medium was removed and replaced with medium containing 50 nM MitoTracker CMXRos. After 20 min of incubation, the MitoTracker-containing medium was removed, and the cells were washed twice with PBS. Finally, the cells were incubated in fresh culture medium before imaging.

### 2.12. Determination of Lipid Peroxidation

The cellular level of lipid reactive oxygen species (ROS) was assessed using the C11 BODIPY581/591 probe (S0043M, Beyotime, Haimen, China) according to the manufacturer’s protocol. After washing with phosphate-buffered saline (PBS), the cells were incubated with 2 µM C11-BODIPY581/591 for 15 min at room temperature in the dark. Fluorescence microscopy was then used to analyze the samples.

### 2.13. Animal Studies

All animal procedures were performed in compliance with the institutional guidelines and were approved by the Animal Care and Welfare Committee of Lanzhou University Second Hospital (Approval No. D2025-659). Female nude mice (6–8 weeks old) sourced from GemPharmatech (Nanjing, China) were subcutaneously injected in the flank with HEC-1-A cells (5 × 10^6^) to generate the xenograft model. Tumor size was measured every two days using a digital caliper, and was calculated using the formula: V = (length × width^2^)/2 (mm^3^). When the tumor volume reached approximately 100 mm^3^, the mice were randomly divided into three groups (*n* = 6 per group) and administered intraperitoneal injections of PBS, S.C (10 mg/kg), or S.C (20 mg/kg) every two days for two weeks. After seven treatment cycles, the mice were euthanized, and the tumors were harvested. The excised tumor tissues were then processed for comprehensive pathological and molecular evaluations, including hematoxylin and eosin (H&E) staining, immunohistochemistry (IHC), and Western blotting analysis.

### 2.14. Histopathologic Analyses

For immunohistochemical (IHC) staining, the excised xenograft tumors were paraffin-embedded and sectioned. Subsequently, the sections were subjected to hematoxylin and eosin (H&E) staining and IHC staining for Ki-67 to assess proliferative activity. All images were captured using an optical microscope (Olympus, Tokyo, Japan).

### 2.15. Statistical Analysis

Statistical analyses were performed using GraphPad Prism software (version 8.0). Data are expressed as the mean ± standard deviation (SD). Differences between groups were assessed by Student’s *t*-test or one-way ANOVA, as appropriate. A *p*-value of less than 0.05 was considered statistically significant.

## 3. Results

### 3.1. S.C Inhibits Proliferation and Invasion in Endometrial Cancer Cells

**Figure 1:**
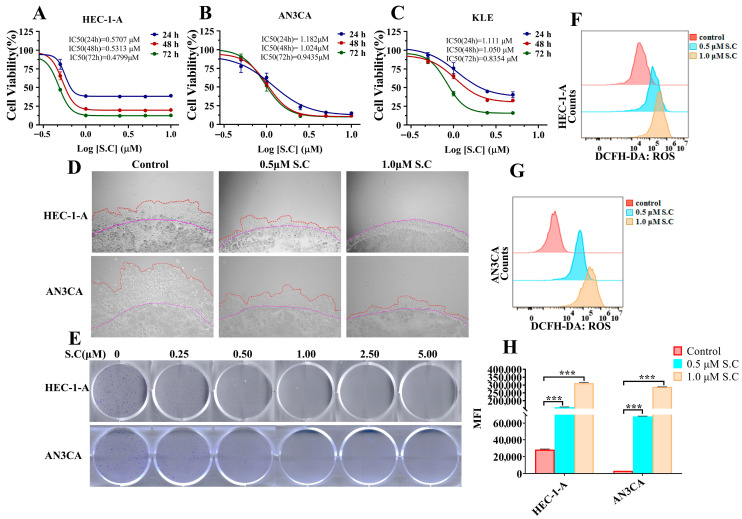
S.C suppresses proliferation and invasion in EC cells. (**A**–**C**) The viability of EC cells treated with specified concentrations of S.C for 24, 48, and 72 h was determined by CCK-8 assay. (**D**) The invasive capacity of EC cells after 4-day S.C treatment was evaluated using a 3D Matrigel drop invasion assay. The edge of the Matrigel drop is outlined with purple dashed lines, and the invasion front of the cells is indicated with red dashed lines. (**E**) Colony formation ability of EC cells exposed to graded concentrations of S.C was examined by crystal violet staining. (**F**–**H**) Intracellular ROS levels in EC cells treated with 0, 0.5, or 1.0 μM S.C for 48 h were quantified by flow cytometry using the fluorescent probe DCFH-DA (10 μM). Data are presented as means ± SD from three independent experiments. *** *p* < 0.001 versus the corresponding control group.

Sanguinarine chloride (S.C), a benzophenanthridine alkaloid isolated from traditional medicinal plants such as *Chelidonium majus*, *Corydalis yanhusuo*, *Sanguinaria canadensis*, and *Macleaya cordata*, has demonstrated antitumor efficacy against a variety of cancers, including breast, gastric, colorectal, and lung cancer. To investigate its role in endometrial cancer, we treated HEC-1-A, AN3CA, and KLE cells with varying concentrations of S.C. The results revealed that S.C suppressed the viability of these endometrial cancer cells in a concentration-dependent manner (Figure 1A–C). Enhanced invasive capability is a critical factor in tumor metastasis [25]. To determine the effect of S.C on the invasiveness of endometrial cancer cells, a 3D Matrigel drop invasion assay was employed. The results demonstrated that S.C significantly suppressed the invasion of HEC-1-A and AN3CA cells (Figure 1D). To further validate the inhibitory effect of S.C on endometrial cancer cell proliferation, a colony formation assay was conducted. The results showed that treatment with S.C significantly suppressed colony formation in HEC-1-A and AN3CA cells (Figure 1E). Increased ROS levels contribute to the anti-tumor effects of various drugs [26,27]. In our study, it was found that S.C stimulation enhanced ROS levels in HEC-1-A and AN3CA cells (Figure 1F–H). Taken together, the results suggest that S.C can inhibit the proliferation, migration, and invasion of endometrial cancer cells, and the upregulation of ROS expression may contribute to its antitumor effects.

### 3.2. S.C Induces Cell Death and Mitochondrial Dysfunction in Endometrial Cancer Cells

**Figure 2:**
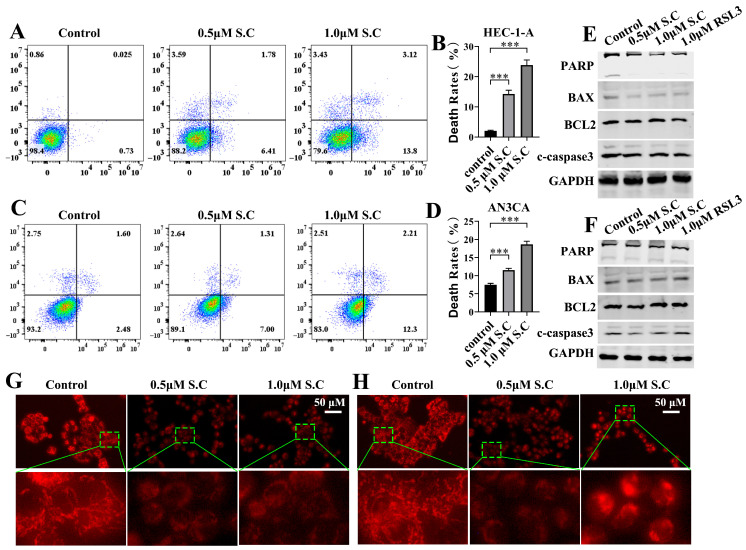
S.C induces cell death and mitochondrial dysfunction in EC cells. (**A**–**D**) Flow cytometric analysis of apoptosis in EC cells treated with S.C (0, 0.5, or 1.0 μM) for 48 h. (**E**,**F**) WB analysis of EC cells treated with the indicated concentrations of S.C (0, 0.5, or 1.0 μM) for 48 h, assessing the protein expression levels of PARP, BAX, BCL2, and c- caspase3. (**G**,**H**) Representative images of EC cells treated with the indicated concentrations of S.C (0, 0.5, or 1.0 μM) for 24 h. Mitochondria were stained with Mito-Tracker Red CMXRos. Data are presented as means ± SD from three independent experiments. *** *p* < 0.001 as compared with corresponding group. cleaved caspase3, c- caspase3.

To investigate whether the anti-tumor effect of S.C on endometrial cancer cells is dependent on the induction of cell death, we performed flow cytometry with Annexin V-FITC/PI staining. As shown in Figure 2A–D, S.C stimulation significantly induced cell death. Apoptosis plays a pivotal role in the mechanism of various anti-tumor drugs. The activation and cleavage of caspase3 act as its key executor, subsequently mediating the cleavage of PARP, among other processes [28]. We analyzed the expression of BAX, BCL2, cleaved caspase3, and PARP in HEC-1-A and AN3CA cells after 48 h of S.C treatment (Figure 2E,F). The results showed that S.C stimulation did not alter the expression of these proteins, suggesting that S.C-induced cell death may differ from apoptosis. In addition to regulating energy supply, the mitochondrion plays a pivotal role in orchestrating cell death through key mechanisms including the regulated release of cytochrome c, the generation of reactive oxygen species (ROS), and the modulation of intracellular calcium homeostasis [29,30,31]. Mitochondrial staining with Mito-Tracker Red CMXRos was performed on HEC-1-A and AN3CA cells following 24 h S.C treatment. We observed a loss of mitochondrial membrane potential, reflected by diminished red fluorescence intensity, along with a reduction in mitochondrial number (Figure 2G,H). This suggests that S.C treatment suppresses mitochondrial function, and the resulting impairment may contribute to S.C-induced cell death.

### 3.3. S.C Provokes Ferroptosis in Endometrial Cancer Cells

**Figure 3:**
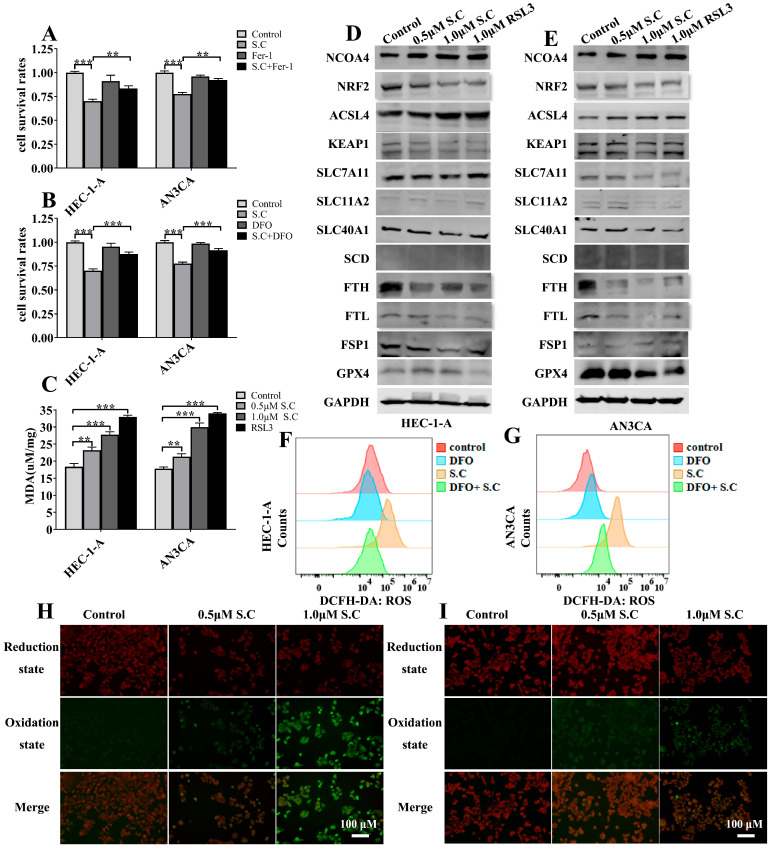
S.C provokes ferroptosis in EC cells. (**A**,**B**) EC cells were treated with S.C (1.0 μM) for 48 h in the presence or absence of Fer-1 (2.0 μM) or DFO (5.0 μM), and cell viability was assessed by CCK-8 assay. (**C**) Intracellular MDA levels were quantified in EC cells treated with S.C or RSL3 (positive control) for 48 h using commercial assay kits. (**D**,**E**) WB analysis of protein levels of NCOA4, NRF2, ACSL4, KEAP1, SLC7A11, SLC11A2, SLC40A1, SCD, FTH, FTL, FSP1, and GPX4 in EC cells treated with S.C for 48 h. (**F**,**G**) Intracellular ROS levels were measured by flow cytometry using the fluorescent probe DCFH-DA (10 μM) in EC cells treated with S.C (1.0 μM) or without DFO (5.0 μM) for 48 h. (**H**,**I**) Lipid peroxidation was detected using C11-BODIPY 581/591 probe after HEC-1-A and AN3CA cells were treated with S.C for 48 h. Red fluorescence represents the reduction state of cells; green fluorescence represents the oxidation state of cells. Data are presented as means ± SD from three independent experiments. ** *p* < 0.01, *** *p* < 0.001 versus the corresponding control group.

Ferroptosis can be driven by several consequences of mitochondrial dysfunction, including elevated ROS, accumulated iron ions, and potentially depleted glutathione [9,10]. To determine whether ferroptosis contributes to S.C-induced cell death, we employed Fer-1 and DFO in our experiments to assess if inhibiting ferroptosis could reverse the suppression of cell viability mediated by S.C. As shown in Figure 3A,B, both Fer-1 and DFO attenuated the suppression of cell viability induced by S.C in HEC-1-A and AN3CA cells. These results suggest that S.C-induced cell death depends on the activation of ferroptosis, which aligns with recent studies indicating that S.C can trigger ferroptosis [13,19,20,21]. Consistent with our previous data, we found that S.C treatment up-regulated the levels of MDA, ROS, and lipid ROS in endometrial cancer cells (Figure 3C,F–I). Given that dysregulation of intracellular free iron, the antioxidant system, and fatty acid metabolism can collectively induce ferroptosis, we sought to investigate whether these pathways contribute to S.C-induced cell death. Our results demonstrated that S.C treatment upregulated the expression of NCOA4 and ACSL4, while downregulating NRF2, GPX4, Ferritin Heavy Chain 1 (FTH) and Ferritin light chain (FTL), in both HEC-1-A and AN3CA cells (Figure 3D,E). Previous studies have established that NCOA4 mediates the autophagic degradation of ferritin (a process known as ferritinophagy) to promote ferroptosis, whereas NRF2 acts as a key transcriptional activator of GPX4 and suppresses ferroptosis. Therefore, our findings suggest that S.C triggers ferroptosis in endometrial cancer cells by concurrently activating NCOA4-mediated ferritinophagy and inhibiting the NRF2-GPX4 antioxidant axis. Furthermore, the observed upregulation of ACSL4, an enzyme critical for synthesizing polyunsaturated fatty acid-phospholipids (PUFA-PLs) that are substrates for lipid peroxidation, may also contribute to this process.

### 3.4. ACSL4 Mediates S.C-Induced Ferroptosis in Endometrial Cancer Cells

**Figure 4:**
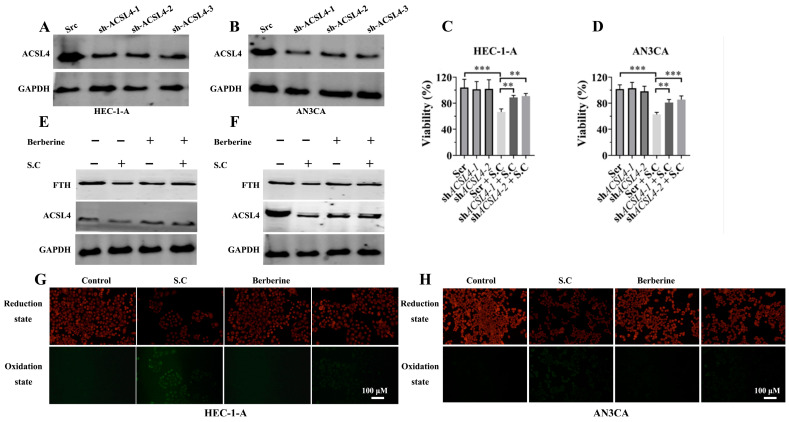
ACSL4 mediates S.C-induced ferroptosis in endometrial cancer cells. (**A**,**B**) Representative WB showing ACSL4 protein levels in the indicated cell lines. (**C**,**D**) The viability of indicated cells following a 48 h treatment with 1.0 μM S.C was determined by the CCK-8 assay. (**E**,**F**) WB analysis of FTH and ACSL4 protein levels in HEC-1-A and AN3CA cells co-treated with 1.0 μM S.C and 30 μM Berberine for 48 h. (**G**,**H**) Lipid peroxidation was assessed in HEC-1-A and AN3CA cells following a 48 h concomitant treatment with 1.0 μM S.C and 30 μM Berberine, using the C11-BODIPY 581/591 probe. Data are presented as means ± SD from three independent experiments. ** *p* < 0.01, *** *p* < 0.001 versus the corresponding control group.

Previous studies have established that sanguinarine chloride can induce ferroptosis by impairing GPX4 activity and promoting intracellular free iron accumulation [13,20,21]. However, whether ACSL4, a key regulator of ferroptosis, is involved in this process remains unreported. Therefore, we aimed to investigate the role of ACSL4 in S.C-induced ferroptosis. First, we knocked down ACSL4 expression in HEC-1-A and AN3CA cell lines (Figure 4A,B) and assessed its impact on S.C-mediated suppression of cell viability. The results showed that ACSL4 knockdown rescued the inhibitory effect on cell viability induced by S.C treatment (Figure 4C,D). Previous studies have shown that berberine binds to and inhibits ACSL4 activity, thereby suppressing ferroptosis. To investigate the effect of ACSL4 inhibition by berberine on S.C-induced ferroptosis, we analyzed relevant markers. Our results demonstrated that berberine upregulates FTH levels and suppresses lipid ROS production in S.C-treated HEC-1-A and AN3CA cells (Figure 4E–H). These results collectively suggest that the upregulation of ACSL4 expression contributes to S.C-induced ferroptosis in endometrial cancer cells.

### 3.5. S.C Suppresses FTO to Upregulate ACSL4 and Promotes Lipid Peroxidation

**Figure 5:**
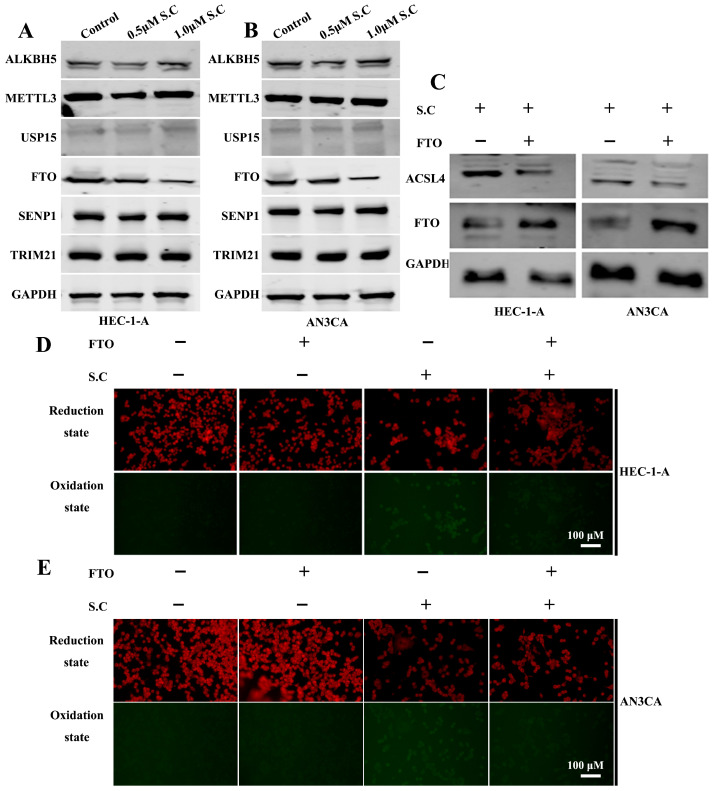
S.C suppresses FTO to upregulate ACSL4 and to promote lipid peroxidation. (**A**,**B**) WB analysis of protein levels of ALKBH5, METTL3, USP15, FTO, SENP1, and TRIM21 in HEC-1-A and AN3CA cells treated with 0, 0.5, or 1.0 μM S.C for 48 h. (**C**) Analysis of ACSL4 and FTO protein expression by WB in FTO-overexpressing cells with or without 1.0 μM S.C treatment. (**D**,**E**) Lipid peroxidation was evaluated in FTO-overexpressing cells following treatment with 1.0 μM S.C or under control conditions, using the C11-BODIPY 581/591 probe.

m6A methylation regulates the stability of ACSL4 mRNA. Methyltransferase 3 (METTL3) has been shown to promote ACSL4 mRNA stability through m6A modification [32]. AlkB homolog 5, RNA demethylase (ALKBH5) enhances both ACSL4 mRNA stability and ferroptosis by binding to the m6A-modified sites at positions 669 and 2015 within the 3′ UTR of ACSL4 mRNA [33]. In contrast, FTO, an m6A demethylase, reduces m6A modification and suppresses ACSL4 expression [34]. In addition, post-translational modifications also regulate the expression of ACSL4 protein in cells. Current research has found that USP15, TRIM21, and SENP1 can modulate the stability of the ACSL4 protein by influencing its ubiquitination and SUMOylation [35,36]. To investigate whether S.C regulates ACSL4 expression through these mechanisms, we analyzed the expression levels of these key factors (Figure 5A,B). The results showed that S.C suppresses FTO protein expression in endometrial cancer cells. To validate the role of FTO in S.C-induced ACSL4 expression, we overexpressed FTO in endometrial cancer cells. The results showed that FTO overexpression suppressed ACSL4 expression in cells stimulated with S.C (Figure 5C). Further, we analyzed cellular lipid peroxidation levels, which demonstrated that FTO overexpression also inhibited lipid peroxidation levels under S.C treatment (Figure 5D,E).

### 3.6. S.C Inhibits Endometrial Tumor Growth by Triggering Ferroptosis In Vivo

**Figure 6:**
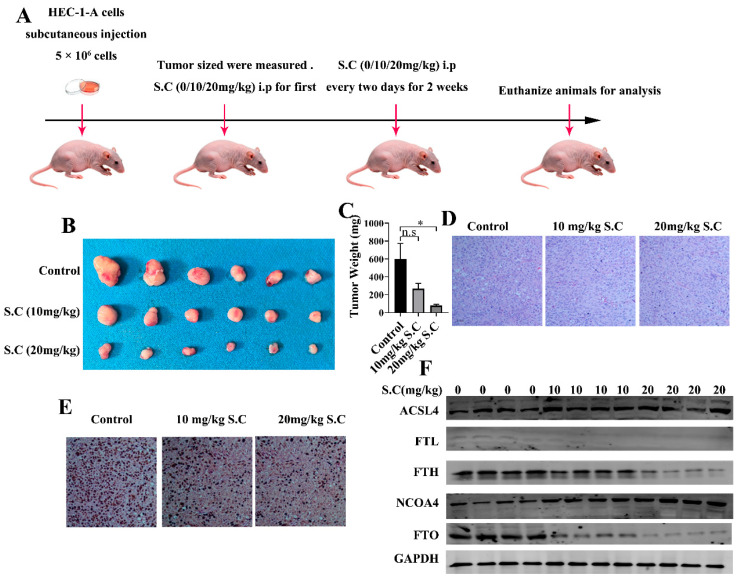
S.C inhibits endometrial tumor growth by triggering ferroptosis in vivo. (**A**) Mice bearing HEC-1-A xenografts were treated intraperitoneally with S.C or PBS once every two days (*n* = 6 in each group) for a total of 14 days. (**B**) Tumors were harvested and photographed. (**C**) Tumor weights were measured and compared across groups. (**D**,**E**) Representative images of H&E staining and IHC staining for Ki67 are presented. (**F**) Protein expression levels of ACSL4, FTH, FTL, NCOA4, NRF2, and GPX4 were assessed by WB analysis. Data are presented as means ± SD. * *p* < 0.05; n.s, not significant.

Previous studies have demonstrated that S.C suppresses tumor growth in various cancers, including prostate and gastric cancer, in vivo [13,16]. However, its role in endometrial cancer progression remains unclear. To address this, we established a subcutaneous xenograft model in nude mice using HEC-1-A cells to evaluate the effect of S.C on endometrial tumor growth (Figure 6A). As shown in Figure 6B,C, S.C treatment significantly inhibited endometrial tumor growth in vivo. Immunohistochemical analysis further revealed a marked reduction in the expression of the proliferation marker Ki67 in the S.C-treated group compared to the control group (Figure 6E). Consistent with the in vitro findings, we observed that S.C treatment downregulated the protein expression of NRF2, GPX4, FTH, and FTL, while enhancing the expression of ACSL4 and NCOA4 in tumor tissues (Figure 6F). These results strongly suggest that S.C induces ferroptosis and suppresses tumor growth in endometrial cancer, both in vitro and in vivo.

## 4. Discussion

Endometrial cancer remains a major clinical challenge in gynecologic oncology. Although patients diagnosed at an early stage generally have favorable outcomes, those with advanced or recurrent disease face limited treatment options and poor survival [3,4]. This stark contrast underscores the pressing need to develop innovative treatment strategies that can overcome conventional therapy resistance. A key obstacle in managing advanced disease lies in cancer cells’ remarkable ability to evade programmed cell death—a phenomenon notably observed in treatment-resistant malignancies [37,38,39]. In this context, ferroptosis has emerged as a promising alternative cell death mechanism. This iron-dependent process, characterized by excessive lipid peroxidation, demonstrates unique potential in eliminating apoptosis-resistant cancer cells [40]. Recent studies have shown that the natural benzophenanthridine alkaloid Sanguinarine Chloride (S.C) acts as a ferroptosis inducer to inhibit tumor growth in various cancer types [13,16,19,20,21]; however, its effects in endometrial cancer have not yet been reported. Our research identifies S.C as a potent ferroptosis inducer in endometrial cancer and reveals a previously unrecognized regulatory axis centered on FTO-ACSL4 signaling, thereby proposing a novel therapeutic approach for this challenging malignancy.

Consistent with its known broad-spectrum anti-tumor properties, our initial experiments confirmed that S.C effectively suppresses key malignant phenotypes of endometrial cancer cells, including proliferation, clonogenic survival, and invasion [16,17,20]. Although S.C has been reported to trigger apoptotic signaling in other cancer types, we found no evidence of apoptosis activation in EC cells. Instead, we demonstrated that S.C inhibits endometrial cancer progression primarily through induction of ferroptosis, as confirmed by the reversal of its cytotoxic and pro-ferroptotic effects upon treatment with the specific ferroptosis inhibitors Fer-1 and DFO. Mechanistically, S.C downregulated the NRF2-GPX4 antioxidant axis and enhanced NCOA4-mediated ferritinophagy, collectively fostering iron accumulation and lipid peroxidation. A central finding was the essential role of ACSL4 in S.C-induced ferroptosis: both genetic knockdown and pharmacological inhibition of ACSL4 restored cell viability and suppressed lipid peroxidation. Further investigation revealed that S.C suppresses FTO expression, leading to increased m6A methylation and mRNA stability of ACSL4, and consequently, elevated ACSL4 protein levels. This epigenetic mechanism was functionally validated by FTO overexpression, which counteracted S.C-induced ACSL4 upregulation, lipid peroxidation, and MDA production. Finally, in vivo studies confirmed that S.C significantly inhibits tumor growth in mouse xenograft models. Tumor tissue analysis recapitulated the downregulation of NRF2-GPX4 and upregulation of ACSL4 and NCOA4, providing strong evidence that the FTO-ACSL4-mediated ferroptosis pathway is operational in vivo and serves as a primary driver of S.C’s anti-tumor efficacy.

While our study delineates a coherent mechanistic pathway, several limitations must be acknowledged. First, the use of a subcutaneous xenograft model does not fully recapitulate the orthotopic tumor microenvironment of the endometrium. Future work should employ orthotopic models to validate these findings in a more physiologically relevant setting, including potential interactions with stromal and immune cells. Second, the precise mechanism by which S.C suppresses FTO protein expression remains unclear. Further investigations are warranted to determine whether S.C promotes FTO degradation or inhibits its transcription.

Building on these findings, several promising research directions emerge. A critical next step is to evaluate whether S.C can synergize with standard chemotherapies such as carboplatin and paclitaxel in endometrial cancer. Given that ferroptosis represents a distinct cell death modality, its combination with apoptosis-inducing agents may prove highly effective against heterogeneous tumors and help overcome chemo-resistance. Additionally, the interplay between ferroptosis and anti-tumor immunity represents a frontier in cancer research. Future studies using immunocompetent mouse models should explore whether S.C-induced ferroptosis can stimulate an immune-responsive TME and potentially synergize with immune checkpoint inhibitors. Finally, exploring other FTO inhibitors and assessing the generalizability of the FTO-ACSL4 axis across other cancer types may hold broad therapeutic implications.

## 5. Conclusions

This study elucidates a previously unrecognized mechanism through which S.C exerts its anti-tumor effects in endometrial cancer. We demonstrate that S.C induces ferroptosis through an FTO-ACSL4 axis: by suppressing FTO expression, S.C enhances ACSL4 expression, thereby increasing cellular sensitivity to lipid peroxidation. This pathway operates in conjunction with suppression of the NRF2-GPX4 antioxidant axis and enhancement of NCOA4-mediated ferritinophagy, creating a synergistic pro-ferroptotic environment. Our findings not only provide insight into the molecular basis of S.C’s anti-cancer activity but also highlight the FTO-ACSL4 axis as a promising therapeutic target for endometrial cancer treatment. The consistent efficacy observed across in vitro and in vivo models underscores the translational potential of targeting this pathway, particularly for aggressive or treatment-resistant endometrial cancers where conventional therapies often fail. Future development of S.C derivatives or other FTO-ACSL4 axis modulators may offer new hope for patients facing this challenging malignancy.

## Data Availability

The raw data supporting the conclusions of this article will be made available by the authors on request.

## Abbreviations

The following abbreviations are used in this manuscript: EC, Endometrial cancer; S.C, Sanguinarine Chloride; WB, Western blotting; MDA, Malondialdehyde; H&E, Hematoxylin and eosin; IHC, Immunohistochemistry; FTO, Fat mass and obesity-associated protein; ACSL4, acyl-CoA synthetase long chain family member 4; ROS, Reactive oxygen species; NCOA4, Nuclear receptor coactivator 4; NRF2, Nuclear factor erythroid derived 2-like 2; GPX4, Glutathione peroxidase 4; Fer-1, Ferrostatin-1; DFO, deferoxamine; BAX, Bcl-2 associated X protein; SLC7A11, Solute carrier family 7 member 11; SLC40A1, Solute carrier family 40 member 1; FTH, Ferritin heavy chain 1; FTL, Ferritin light chain; FSP1, Ferroptosis suppressor protein 1; SLC11A2, Solute carrier family 11 member 2; SCD1, Stearoyl-CoA desaturase 1; ALKBH5, AlkB homolog 5, RNA demethylase; METTL3, Methyltransferase 3; USP15, Ubiquitin specific peptidase 15; SENP1, SUMO Specific Peptidase 1; TRIM21, Tripartite motif containing 21; KEAP1, Kelch-like ECH-associated protein 1; BCL2, B-cell lymphoma/leukemia-2 gene; PARP, Poly ADP-ribose polymerase; CFU, Colony-forming unit; BCA, Bicinchoninic acid; PBS, Phosphate-buffered saline; MFI, Mean fluorescence intensity; PUFA-PLs, Polyunsaturated fatty acid-phospholipids.
